# Coexisting Thalassemia, Immune Thrombocytopenic Purpura, and Multiple Myeloma With Osteopenia: Complex Hematologic Case in an Adult Asian Male Patient

**DOI:** 10.7759/cureus.75338

**Published:** 2024-12-08

**Authors:** Javier B Chambi-Torres, Sohair Angly, George Michel

**Affiliations:** 1 Internal Medicine, Larkin Community Hospital, South Miami, USA

**Keywords:** corticosteroid therapy, diagnosis of multiple myeloma, immune thrombocytopenia purpura, non-transfusion-dependent thalassemia, rituximab

## Abstract

We report a rare case of a 45-year-old Asian male patient with concurrent multiple myeloma (MM), immune thrombocytopenic purpura (ITP), and thalassemia trait, presenting with severe thrombocytopenia, back pain, and bleeding manifestations. The diagnosis was established through a combination of laboratory findings, imaging, and bone marrow biopsy, revealing 90% plasma cell involvement and KRAS/BRCA2 mutations. Management focused on controlling ITP with corticosteroids, rituximab, and platelet transfusions while addressing immunosuppression risks. Due to logistical limitations, MM-specific therapy was deferred, and the patient was stabilized for transfer to continue treatment in his home country. This case highlights the challenges of managing overlapping hematologic disorders and underscores the importance of individualized care in complex presentations.

## Introduction

Multiple myeloma (MM) and thalassemia are hematological disorders that are related to bone involvement [1], and the coexistence of both disorders has been rarely described in the literature [2]. Additionally, a few reports of MM and immune thrombocytopenic purpura (ITP) were described in the literature too [3]. This simultaneous occurrence of these conditions presents significant clinical challenges due to overlapping symptoms and the potential for severe complications.

Thalassemia is a genetic blood disorder causing anemia due to reduced hemoglobin production, and it includes alpha-thalassemia (gene deletions) and beta-thalassemia (gene mutations), ranging from mild to severe forms that may require lifelong transfusions [4]. Meanwhile, MM is a clonal plasma cell disorder marked by abnormal monoclonal paraprotein increase, leading to end-organ damage such as hypercalcemia, renal dysfunction, anemia, and bone lesions [5]. ITP, an autoimmune disorder characterized by low platelet count, purpura, and bleeding due to antiplatelet autoantibodies, involves platelet destruction by spleen macrophages and it is a diagnosis of exclusion that requires immunosuppression and splenectomy sometimes [6].

The coexistence of these conditions complicates the clinical picture and poses diagnostic and therapeutic challenges. This case report aims to contribute to the limited literature on the coexistence of MM, thalassemia, and ITP, providing insights into their management and emphasizing the importance of a comprehensive diagnostic approach.

## Case presentation

A 45-year-old Asian male cruise-line worker with thalassemia trait, ITP, and hypertension presented with thrombocytopenia, easy bruising, mid-thoracic back pain, recent painless hematuria, and intermittent epistaxis. Diagnosed with ITP in 2016, he had previously responded to prednisone. On examination, he had ecchymosis and petechiae distributed in both arms and legs. Laboratory findings on admission (Table 1) at the hospital revealed a platelet count of 19 x 10^3/μL, total protein of 10.6 g/dL, globulin of 6.7 g/dL, hemoglobin of 10.3 g/dL, mean corpuscular volume (MCV) of 63 fL, elevated Immunoglobulin G (IgG) of 7097 mg/dL, decreased Immunoglobulin A (IgA) of 16 mg/dL and Immunoglobulin M (IgM) of 11 mg/dL. Finally, M-spike on serum protein electrophoresis (SPEP) resulted in 4.4 g/dL. 

**Table 1:** Laboratory findings and reference range. MCV: mean corpuscular volume.

Laboratory test	Result	Reference range
Platelet count	19 x 10^3/μL	150-450 x 10^3/μL
Total protein	10.6 g/dL	6.0-8.1 g/dL
Globulin	6.7 g/dL	2.4-3.5 g/dL
Hemoglobin	10.3 g/dL	12.1-16.1 g/dL
MCV	63 fL	79.0-92.2 fL
IgG	7097 mg/dL	603-1613 mg/dL
IgA	16 mg/dL	90-386 mg/dL
IgM	11 mg/dL	20-172 mg/dL

**Figure 1:**
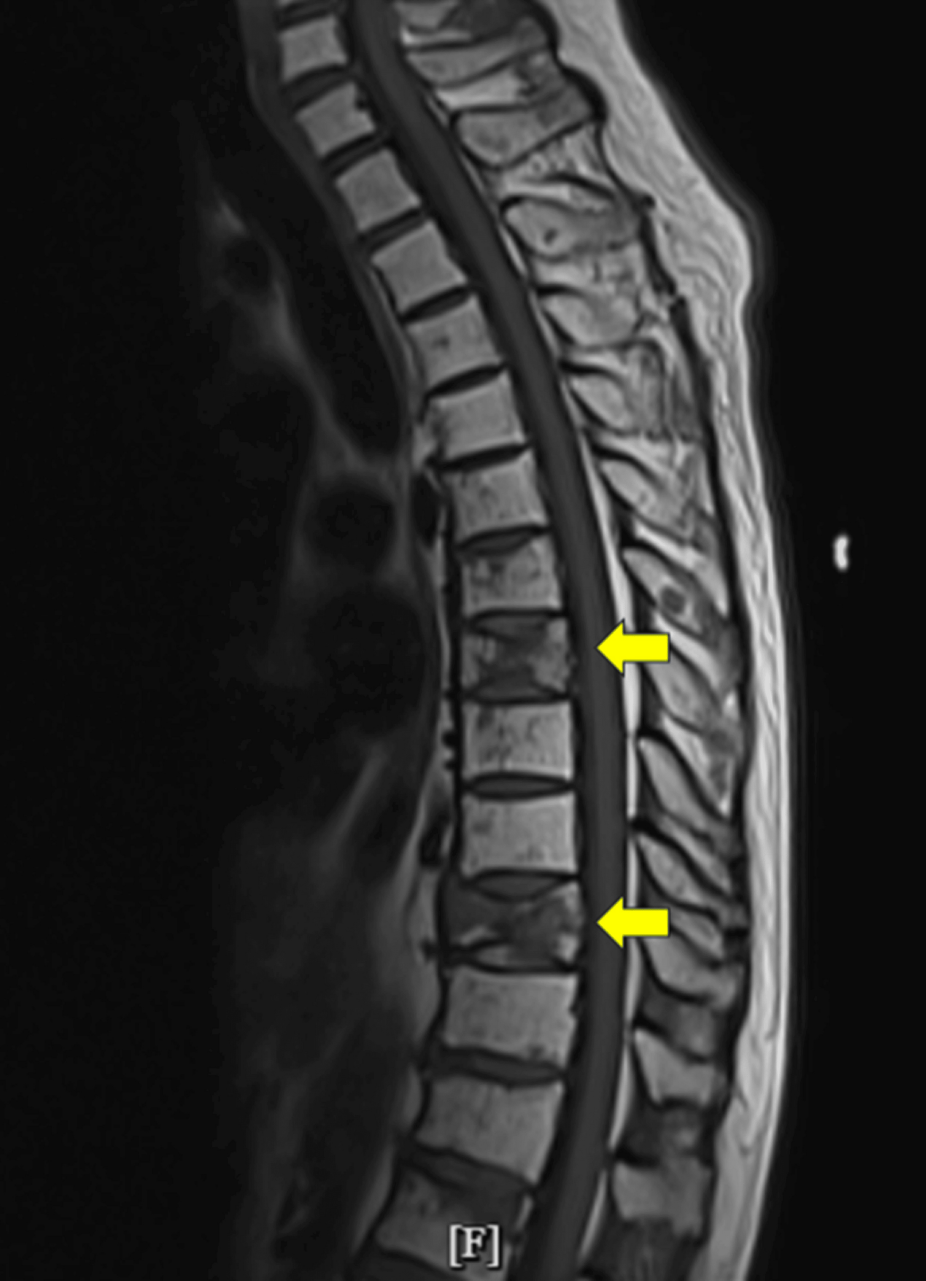
Findings suggestive of metastatic disease involving the thoracic spine with the largest lesion being in the T10 vertebral body. Pathologic compression fractures T7 and T10 (yellow arrows).

Imaging showed diffuse osteopenia with T7 and T10 pathologic fractures, suggestive of metastatic disease or MM involvement, confirmed by magnetic resonance imaging (MRI) (Figure 1). 

**Figure 2:**
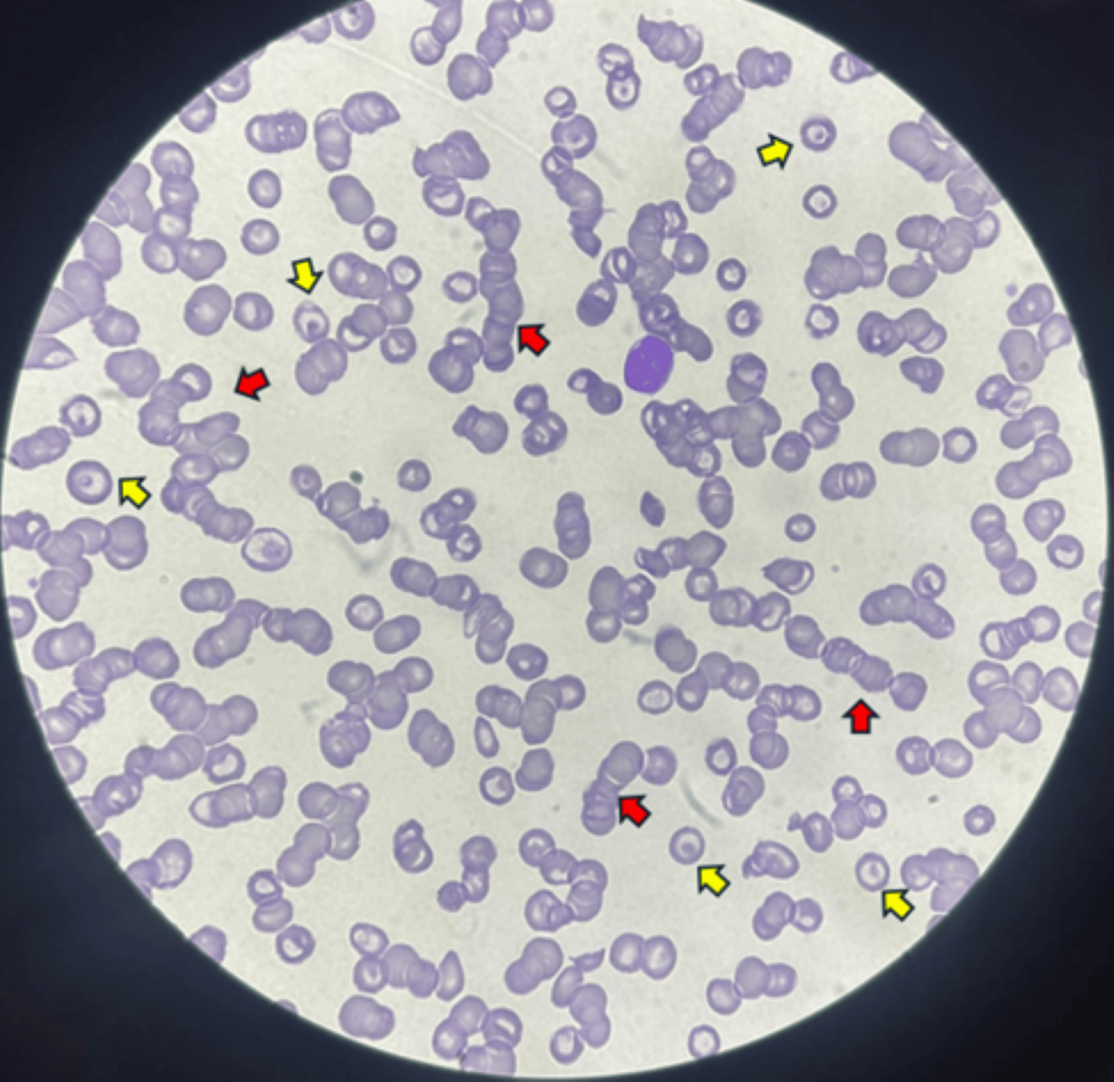
Peripheral blood smear: Rouleaux formation (red arrow) and target cells (yellow arrows).

Peripheral smear exhibited rouleaux formation, target cells, and low platelets (Figure 2). HIV, *Helicobacter pylori*, and antinuclear antibody (ANA) tests were negative.

**Figure 3:**
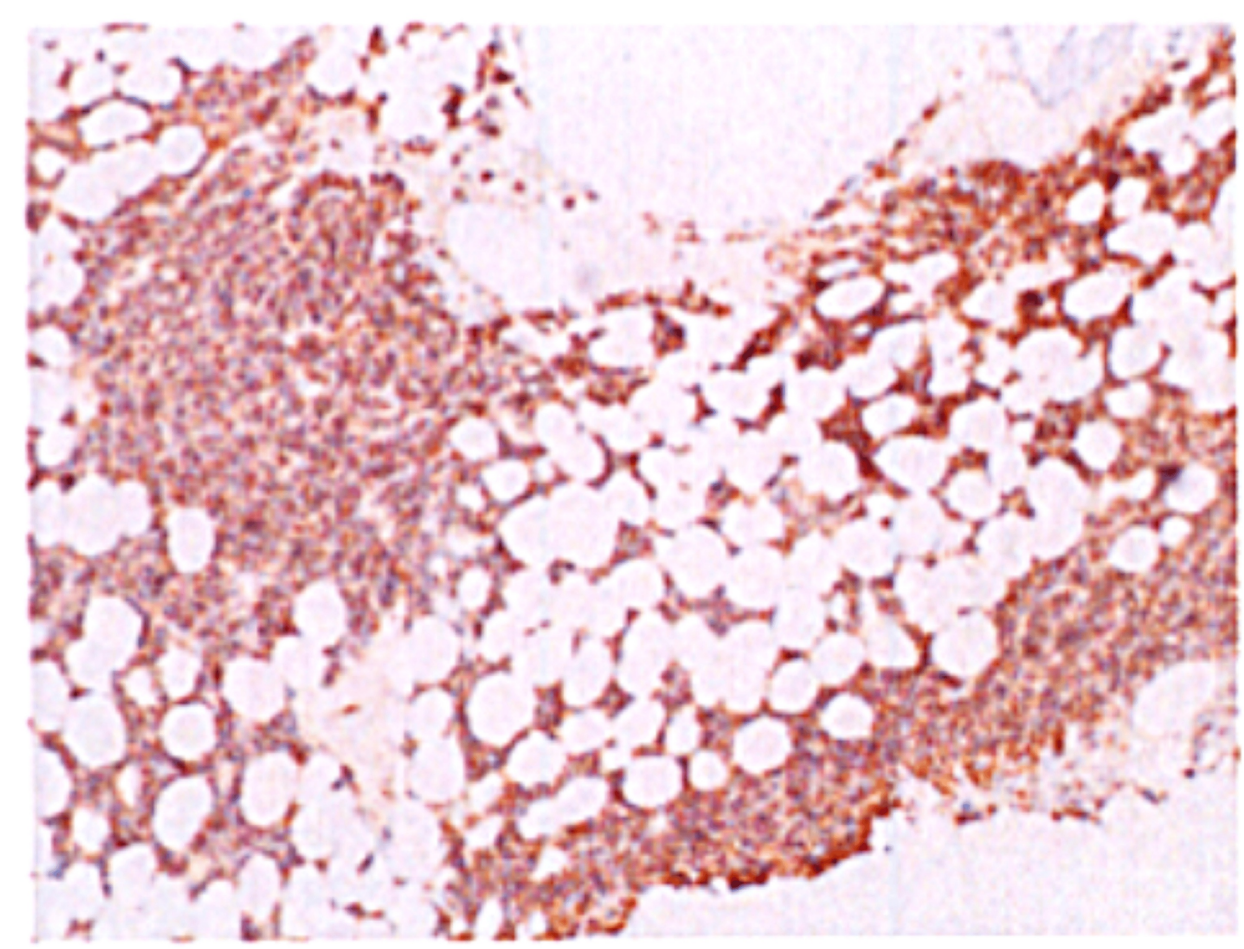
Plasma cell neoplasm involving 90% of bone marrow cellularity.

Bone marrow biopsy revealed 90% plasma cell neoplasm (Figure 3), kappa-restricted monoclonal plasma cells, and 5.8% positivity for CD19, CD38, CD56, CD117, CD138, and c-kappa. KRAS and BRCA2 mutations were found by using next-generation sequencing (NGS). Elevated erythrocyte sedimentation rate (ESR) and reduced IgA and IgM were notable.

The patient was treated with dexamethasone 40 mg intravenous for four days, platelet transfusions, and romiplostim to manage thrombocytopenia, which fluctuated significantly due to ITP and marrow suppression from MM. Despite multiple transfusions, his platelet counts remained low due to severe shortages at the blood bank. The patient stated that he wanted to continue further evaluation and management in the Philippines (his home country) because he wanted to have the support of his family. Then he received rituximab, which improved his platelet count to 40 x 10^3/μL, enabling travel back to the Philippines for MM treatment. During hospitalization, he was counseled on infection risks due to immunosuppression and the bleeding risks associated with his low platelet count. The patient tolerated his therapies well, including rituximab, with only mild transient headache and no signs of acute distress upon discharge.

## Discussion

The coexistence of MM, thalassemia, and ITP is extremely rare and complex, with limited cases reported in the literature. Managing these overlapping hematologic disorders is challenging, as the treatment for one condition may exacerbate the symptoms of the others. Reports indicate that patients with MM and thalassemia, for instance, often face increased risks of hematologic malignancies and iron overload due to recurrent blood transfusions [2]. Additionally, the occurrence of MM and ITP has been documented as an uncommon but noteworthy association [7], often requiring a delicate balance in treatment due to bleeding risks associated with ITP and the myelosuppressive effects of MM therapy.

This patient’s case is further complicated by mutations in KRAS and BRCA2 genes within plasma cells. The KRAS gene, frequently mutated in MM, is known to activate signaling pathways like MEK/ERK and PI3K/AKT, contributing to tumor cell survival and proliferation [8]. Studies have shown that while KRAS mutations are common in MM, the impact on survival varies, with some reports indicating that the activation of the MAPK pathway via the KRAS expression correlates with poor prognosis [9]. The BRCA2 mutation, associated with impaired DNA repair, further exacerbates genomic instability in MM, potentially leading to faster disease progression [10].

The NCCN (National Comprehensive Cancer Network) guidelines recommend a comprehensive diagnostic approach for multiple myeloma, including SPEP, immunofixation, and bone marrow biopsy, supported by imaging for bone involvement [11]. In our case, the diagnosis was confirmed through SPEP revealing an M-spike of 4.4 g/dL, bone marrow biopsy demonstrating 90% plasma cell infiltration with KRAS and BRCA2 mutations, and MRI identifying pathologic fractures at T7 and T10. ITP in MM patients also complicates the diagnostic process as thrombocytopenia could initially be mistaken for marrow failure due to MM itself [12]. Initial management included dexamethasone to address ITP-related thrombocytopenia and suppress plasma cell activity. However, our approach deviated from NCCN's preferred use of proteasome inhibitors, such as bortezomib, due to the patient's severe thrombocytopenia and bleeding risks. Instead, we prioritized stabilizing platelet counts with rituximab and romiplostim, enabling safe discharge for further treatment abroad. This tailored management highlights the necessity of adapting guideline recommendations to complex, multifactorial presentations. Comprehensive evaluation, including genetic testing and advanced imaging, is critical for accurately diagnosing the contributions of each condition to the overall clinical picture.

Treatment for this patient required a multifaceted approach. Corticosteroids, platelet transfusions, and lastly, rituximab were used to manage ITP, effectively increasing platelet counts. However, prolonged corticosteroid use is concerning in MM due to adverse effects on bone health and increased infection risks. In MM with KRAS gene mutations, future directions of targeting the MEK/ERK and PI3K/AKT pathways presents a promising therapeutic approach, and additionally, receptor tyrosine kinase inhibitors, such as those targeting AXL, and immunotherapies like CAR T-cell therapy are being explored to address drug resistance and improve treatment outcomes [13]. In multiple myeloma cases with BRCA mutations, therapies targeting impaired DNA repair pathways, such as poly(ADP-ribose) polymerase (PARP) inhibitors, may offer promising results. Studies have shown that agents like PJ34 can enhance cytotoxicity and promote apoptosis when combined with alkylating agents, effectively overcoming drug resistance by exploiting the weakened DNA repair mechanism [14], but more studies are needed.

## Conclusions

This case illustrates the complexities of managing a patient with coexisting multiple myeloma, thalassemia, and ITP, where overlapping symptoms and multifactorial thrombocytopenia posed significant diagnostic and therapeutic challenges. The patient was treated with prednisone for ITP, supportive care for thalassemia, and planned chemotherapy for MM. However, logistical difficulties arose as the patient expressed a strong desire to return home, complicating the initiation of advanced therapies. This case emphasizes the importance of personalized, patient-centered care and the need for flexible management strategies in resource-limited or transitional healthcare settings.
